# Taste Dysfunction in Oral Candidiasis: Impact of *Candida* Carriage and Hyphal Presence

**DOI:** 10.1111/myc.70145

**Published:** 2026-02-20

**Authors:** Younjung Park, Hyo‐Jung Jung, Sin Hye Hwang, Eunae Sandra Cho, Hyung‐Joon Ahn

**Affiliations:** ^1^ Department of Orofacial Pain and Oral Medicine Yonsei University College of Dentistry Seoul Republic of Korea; ^2^ Department of Orofacial Pain and Oral Medicine, Oral Science Research Institute Yonsei University College of Dentistry Seoul Republic of Korea; ^3^ Department of Periodontology Gangnam Severance Dental Hospital Seoul Republic of Korea; ^4^ Department of Oral Pathology, Oral Cancer Research Institute Yonsei University College of Dentistry Seoul Republic of Korea

**Keywords:** antifungal agents, biofilms, *Candida albicans*, fluconazole, hyphal morphogenesis, oral candidiasis, saliva, taste disorders

## Abstract

**Background:**

Taste dysfunction is a common symptom of oral candidiasis; however, its underlying mechanisms remain unclear.

**Objectives:**

This study aimed to determine whether *Candida albicans* carriage, particularly its hyphal phenotype, is associated with impaired taste sensitivity.

**Patients/Methods:**

Fifty‐seven participants were divided into three groups: noncarriers (*n* = 20), carriers without hyphae (*n* = 20), and carriers with hyphae (*n* = 17). Tongue biofilm samples were collected for *Candida* culture and smear tests. Salivary flow rates were measured, and taste sensitivity was assessed using standardised taste strips. Carriers underwent topical fluconazole treatment, and post‐treatment taste sensitivity was re‐evaluated.

**Results:**

Carriers exhibited reduced sensitivity to sweet and bitter tastes, with an additional decline in umami sensitivity among those with hyphae. In addition, both unstimulated and stimulated salivary flow rates were significantly lower in *Candida* carriers than in noncarriers. Topical fluconazole treatment resulted in a marked reduction in hyphae and significant improvement in taste sensitivity.

**Conclusions:**

This study revealed differences in salivary flow and taste sensitivity between noncarriers and Candida albicans carriers. The presence of hyphal forms was strongly associated with greater impairment in taste function. Improvement following antifungal therapy suggests that hyphal invasion may play a key role in the pathogenesis of taste dysfunction in oral candidiasis.

## 1 Introduction

Candida is a dimorphic fungus that exists in the oral cavity either as a commensal yeast or in an invasive hyphal form [1, 2]. The hyphal phenotype is associated with increased virulence, epithelial infiltration, tissue damage, and biofilm formation [3, 4, 5]. Oral candidiasis (OC) assessments have conventionally focused on overall *Candida* carriage or cultured specimens under controlled conditions [6, 7, 8, 9]. However, recent evidence suggests that biofilm formation plays a critical role in the pathogenesis of OC by creating a persistent, drug‐resistant microbial ecosystem composed of *Candida*, extracellular matrix, and occasionally bacteria [10, 11, 12, 13, 14].

OC is typically classified based on its clinical manifestations, such as pseudomembranous and erythematous candidiasis; however, it often presents with less obvious symptoms, including taste dysfunction [15, 16]. Despite being a hallmark symptom, the precise mechanisms underlying taste impairment in OC remain poorly understood [17, 18]. As the tongue serves as the primary reservoir for *Candida* colonisation, and saliva is essential for transporting taste stimuli, disturbances in these factors may contribute to altered gustatory function [17, 19].

In this study, we investigated the association between *Candida albicans* ( *C. albicans* ) carriage, with an emphasis on its hyphal phenotype, and taste dysfunction and concomitant changes in salivary flow. By employing both *Candida* carriage and smear tests to assess tongue biofilms, we aimed to elucidate the clinical relevance of *Candida* biofilms and hyphal transitions in the development of taste impairment in patients with OC.

## 2 Patients and Methods

### 2.1 Sample Size Calculation

The sample size required for this study was determined using *GPower 3.1.9.7*. A one‐way ANOVA was used as the primary analysis to detect differences among the three groups in taste threshold and related variables. The expected effect size (f = 0.471) was derived from the mean taste threshold values reported by Sakashita et al. [18]. With an effect size of 0.471, a significance level (α) of 0.05, and a statistical power of 85%, the minimum required sample size was calculated to be 54 participants. Considering an anticipated dropout rate of 10%, the final target sample size was set at 60 participants (20 per group).

### 2.2 Participants and Study Design

This prospective clinical study was conducted at Yonsei University Dental Hospital from February 2021 to September 2022, enrolling patients who visited the Department of Orofacial Pain and Oral Medicine with suspected oral candidiasis.

The inclusion criteria were: (1) age between 20 and 65 years; (2) clinical suspicion of oral candidiasis; (3) completion of both candida swab and fungus smear tests; and (4) voluntary written informed consent. The exclusion criteria were: (1) uncontrolled systemic diseases (e.g., diabetes mellitus, gastroesophageal reflux disease, chronic hepatic or renal disease, hypothyroidism); (2) current use of antipsychotic medications; (3) history of Sjögren's syndrome; (4) prior head and neck radiotherapy; (5) significant oral lesions (widespread dental caries, severe periodontal disease with probing depth ≥ 6 mm, stomatitis, or mucosal diseases such as lichen planus); and (6) other reasons deemed inappropriate for participation by the principal investigator.

Eligible participants were classified into three groups according to candida swab and fungus smear test results. For Group 1 (non‐carriers) and Group 2 (carriers without hyphae), random selection among eligible subjects was performed to achieve a target sample size of 20 per group, and additional recruitment ceased once enrollment targets were met. For Group 3 (carriers with hyphae), detection was rare, so recruitment continued until the target was reached among all screened patients. After excluding three subjects in Group 3 who had previously received antifungal agents, a total of 57 participants were included in the final analysis.

The three study groups were defined as follows:

Non‐carriers: negative for both *C. albicans* colonies on swab and hyphal forms on smear (*n* = 20).

Carriers: positive for *C. albicans* colonies on swab but negative for hyphal forms on smear (*n* = 20).

Carriers with hyphae: positive for both *C. albicans* colonies on swab and hyphal forms on smear (*n* = 17).

**FIGURE 1:**
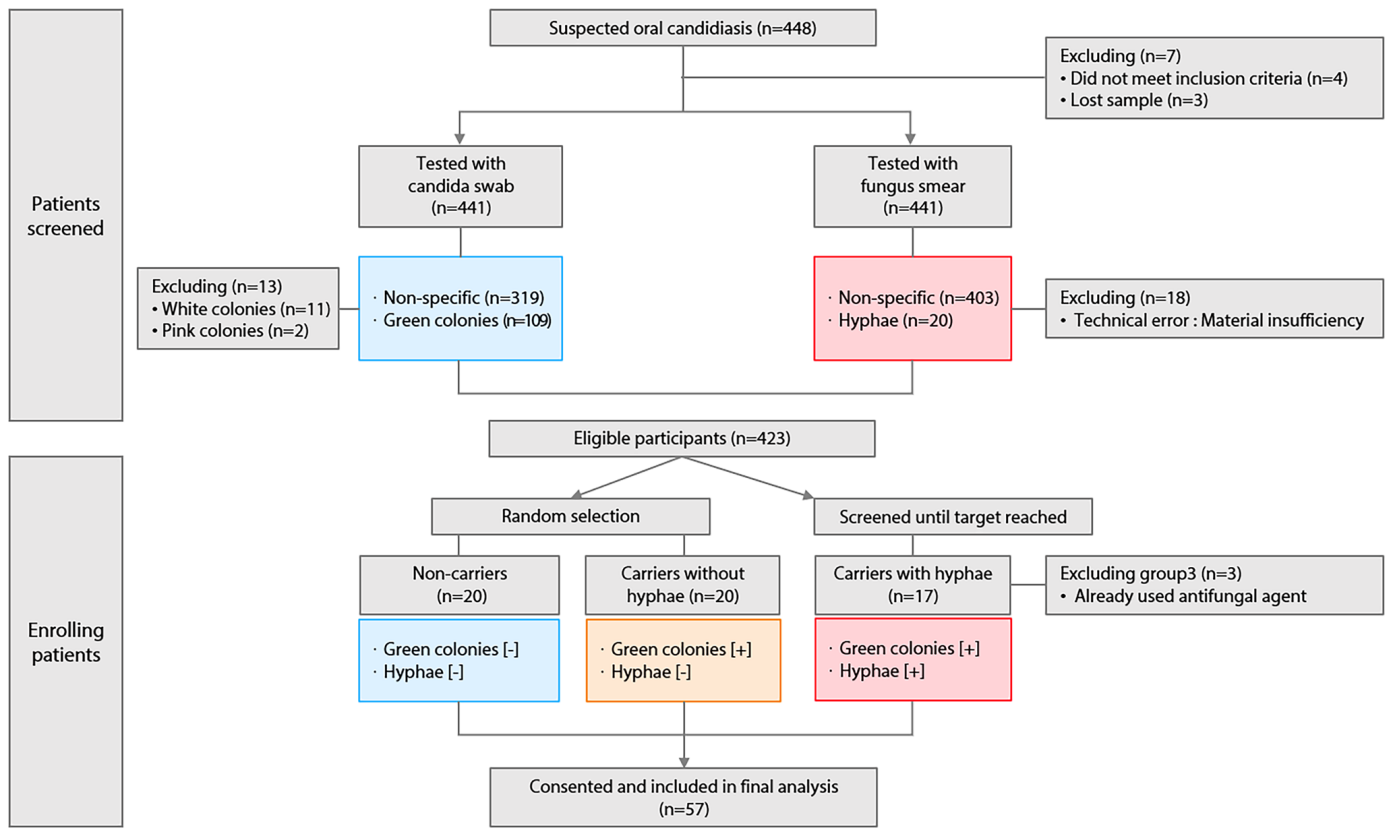
Flow diagram of participant enrollment and group allocation. The study screened 448 adult patients with suspected oral candidiasis. After candida swab and fungus smear testing, exclusions were made as indicated. Eligible participants were classified into three groups according to test results: Non‐carriers (green colonies [−], hyphae [−]), carriers without hyphae (green colonies [+], hyphae [−]), and carriers with hyphae (green colonies [+], hyphae [+]). Random selection was performed for non‐carriers and carriers without hyphae; recruitment for carriers with hyphae continued until the target was reached. Three subjects in the carriers with hyphae group were excluded due to prior antifungal agent use. Ultimately, 57 patients consented and were included in the final analysis.

A detailed flow of participant inclusion, exclusion, and classification is depicted in Figure 1.

### 2.3 Candida Swab Test

**FIGURE 2:**
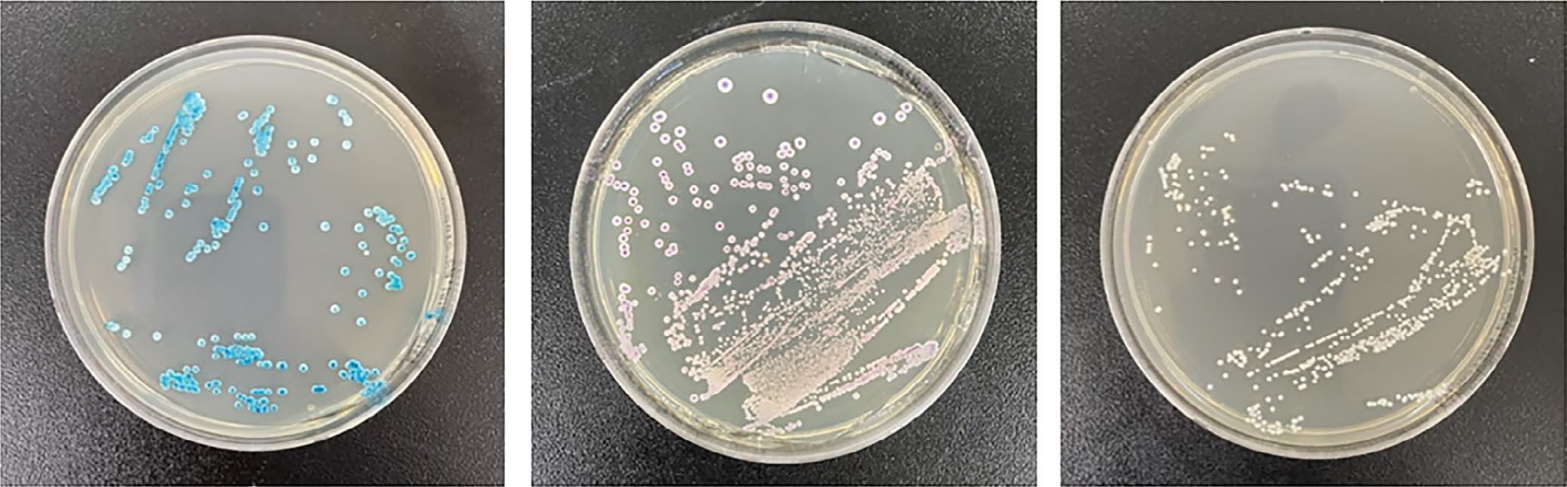
Representative CHROMagar *Candida* culture plates. Photographs illustrating the results of CHROMagar Candida after 48 h of incubation at 37°C. Green colonies indicate the presence of *Candida albicans* (left plate), pink colonies indicate *Candida krusei* (middle plate), and white colonies (right plate) lack specific identification and were excluded from analysis.

To detect *C. albicans* , the biofilm on the participant's tongue dorsum was sampled three times with a sterile cotton swab. The collected sample was then transferred to a liquid transport medium prepared with Difco YM Broth 271,120 (BD DIFCO, USA) according to the manufacturer's instructions and incubated at 37°C for 24 h. Subsequently, 50 μL of the cultured suspension was inoculated onto CHROMagar *Candida* plates (Paris, France) and incubated at 37°C for 48 h. Soft, convex, green colonies were identified as *C. albicans* (Figure 2). Participants were classified as non‐carriers if no green colonies formed and as carriers if green colonies were present [20, 21].

### 2.4 Fungus Smear Test

Participants who tested positive for *C. albicans* in the swab test underwent a fungus smear test for pathological evaluation. A sample was collected from the biofilm on the dorsal surface of the tongue using a wooden tongue depressor and transferred onto a glass slide. It was then fixed with ethyl alcohol and stained using the periodic acid–Schiff (PAS) method. Slides were examined under a light microscope to detect *Candida* hyphae [22]. According to the pathology reading recorded in the electronic medical record (EMR), participants were classified into carriers without hyphae or carriers with hyphae based on the presence or absence of hyphal forms.

### 2.5 Taste Strip Test

Taste sensitivity was assessed using validated taste strips (Burghart, Wedel, Germany) that are widely used in clinical settings. Each set contained five taste qualities (sweet, bitter, salty, sour, and umami), each present at four different concentrations (Table 1), along with two blank (tasteless) strips, yielding a total of 22 test strips. The test was performed according to the manufacturer's instructions. One point was awarded for each correct taste identification, with a maximum score of 20 [23].

**TABLE 1:** Concentrations of the taste strips.

Taste modality	Concentration level			
Sweet (sucrose, g/mL)	0.05	0.1	0.2	0.4
Bitter (quinine‐hydrochloride, g/mL)	0.0004	0.0009	0.0024	0.006
Salty (sodium chloride, g/mL)	0.016	0.04	0.1	0.25
Sour (citric acid, g/mL)	0.05	0.09	0.165	0.3
Umami (monosodium glutamate, g/mL)	0.016	0.04	0.1	0.25

### 2.6 Participant Questionnaire

A structured questionnaire was used to assess patients' subjective oral symptoms, especially tongue symptoms such as spontaneous pain, stimulated pain, and oral dryness; responses for each item were recorded as “yes” or “no”.

### 2.7 Saliva Flow Rate

To exclude participants with xerostomia that could affect taste function, salivary flow rates were assessed at baseline in all subjects. Unstimulated whole saliva (UWS) flow rate was measured by instructing the participant to sit comfortably and tilt their head slightly forward, allowing saliva to naturally pool in the mouth without swallowing. Over a 5‐min period, all spontaneously secreted saliva was expectorated into a pre‐weighed sterile container. The total volume was recorded, and flow rate was expressed in mL/min. Stimulated whole saliva (SWS) flow rate was measured immediately after UWS collection. Participants were asked to chew a standardised, colourless, and odourless gum base at a rate of one chew per second for 5 min, expectorating all saliva into a separate pre‐weighed sterile container. SWS flow rate was calculated in the same way as UWS.

### 2.8 Tongue Moisture

Tongue moisture was assessed using a mucus device (Saitama, Japan). The sensor was placed at the centre of the tongue, approximately 10 mm from the tip, and three readings were recorded. The median of these readings was recorded as the final moisture level.

### 2.9 Antifungal Treatment

In both the “carriers without hyphae” and “carriers with hyphae” groups, where *C. albicans* was detected, a topical fluconazole oral suspension was prescribed for 2 weeks. The concentration (0.2%), frequency (once daily), and amount (5 mL) of the fluconazole application were specified, ensuring the treatment regimen was clearly described and standardised for all participants. All previously administered tests were repeated during the second visit to assess treatment efficacy.

### 2.10 Statistical Analysis

One‐way analysis of variance (ANOVA) and chi‐square tests were used to compare participant characteristics among the groups. Differences in taste sensitivity were analysed using ANOVA followed by Tukey's post hoc test. Changes in participant characteristics following antifungal treatment were summarised using descriptive statistics. Paired *t*‐tests were used to assess within‐group changes in taste sensitivity following antifungal treatment, and independent t‐tests were used for between‐group comparisons. All analyses were performed using SPSS version 25.0 (IBM Corp., Armonk, NY), with statistical significance set at *p* < 0.05.

## 3 Results

### 3.1 Participant Characteristics

A total of 57 participants were included in the study: (1) non‐carriers of *C. albicans* (*n* = 20), (2) carriers without hyphae (*n* = 20), and (3) carriers with hyphae (*n* = 17). Although the mean age was higher in the group with hyphae, the difference was not significant. The clinical tongue symptoms (spontaneous pain, stimulated pain, and subjective dry mouth) were evenly distributed among the groups. The unstimulated and stimulated salivary flow rates and tongue moisture measurements were significantly lower in the carrier groups than in the non‐carrier group (Table 2).

**TABLE 2:** Demographic and clinical characteristics of participants across the three groups.

Variable	Non‐carrier (*N* = 20)	Carriers without hyphae (*N* = 20)	Carriers with hyphae (*N* = 17)	*p*
Age (years)	46.7 ± 11.7	48.9 ± 9.7	54.2 ± 8.6	0.080^†^
Sex				
Male	2 (10.0)	5 (25.0)	2 (11.8)	0.370^‡^
Female	18 (90.0)	15 (75.0)	15 (88.2)	
Spontaneous pain				
Yes	14 (70.0)	8 (40.0)	8 (47.1)	0.141^‡^
No	6 (30.0)	12 (60.0)	9 (52.9)	
Stimulated pain				
Yes	14 (70.0)	12 (60.0)	13 (76.5)	0.552^‡^
No	6 (30.0)	8 (40.0)	4 (23.5)	
Subjective dry mouth				
Yes	9 (45.0)	13 (65.0)	13 (76.5)	0.135^‡^
No	11 (55.0)	7 (35.0)	4 (23.5)	
UWS flow rate	0.31 ± 0.17	0.21 ± 0.11	0.21 ± 0.16	0.045^†^
SWS flow rate	1.32 ± 0.79^a^	0.82 ± 0.33^b^	0.81 ± 0.48^b^	0.011^†^
Tongue moisture	27.4 ± 3.4^a^	25.2 ± 2.1^b^	23.9 ± 2.8^b^	0.001^†^

Abbreviations: SWS, stimulated whole saliva; UWS, unstimulated whole saliva.

^†^ One‐way ANOVA, Mean ± standard deviation, the different letters denote significant differences between the groups by Tukey post hoc analyses.

^‡^ Chi‐square test, N (%).

### 3.2 Taste Sensitivity

Significant differences in taste sensitivity were observed among the groups for sweet (*p* = 0.016), bitter (*p* < 0.001), and umami (*p* = 0.029) tastes. Compared with the non‐carrier group, the carrier groups showed reduced sensitivity to sweet and bitter tastes. Notably, sensitivity to umami taste significantly decreased only in the carrier group with hyphae. Overall, the carrier groups had lower taste sensitivity scores, indicating a general decrease in taste sensitivity (Table 3).

**TABLE 3:** Mean differences in taste sensitivity across the three groups.

Variable	Non‐carrier (*N* = 20)	Carriers without hyphae (*N* = 20)	Carriers with hyphae (*N* = 17)	*p*
Sweet	3.50 ± 0.69^a^	2.80 ± 1.06^b^	2.71 ± 0.92^b^	0.016
Sour	2.25 ± 0.64	1.90 ± 0.64	1.82 ± 0.95	0.178
Salty	2.55 ± 1.19	2.10 ± 1.07	1.94 ± 1.09	0.230
Bitter	3.65 ± 0.67^a^	2.00 ± 1.49^b^	2.00 ± 1.62^b^	< 0.001
Umami	2.50 ± 1.05^a^	2.10 ± 1.45^a^	1.41 ± 1.06^b^	0.029
Total score	14.55 ± 2.12^a^	10.85 ± 3.94^b^	9.82 ± 3.34^b^	< 0.001

*Note:* One‐way ANOVA, Mean ± standard deviation, the different letters denote significant differences between the groups by Tukey post hoc analyses.

### 3.3 Therapeutic Effect of Antifungal Treatment

**FIGURE 3:**
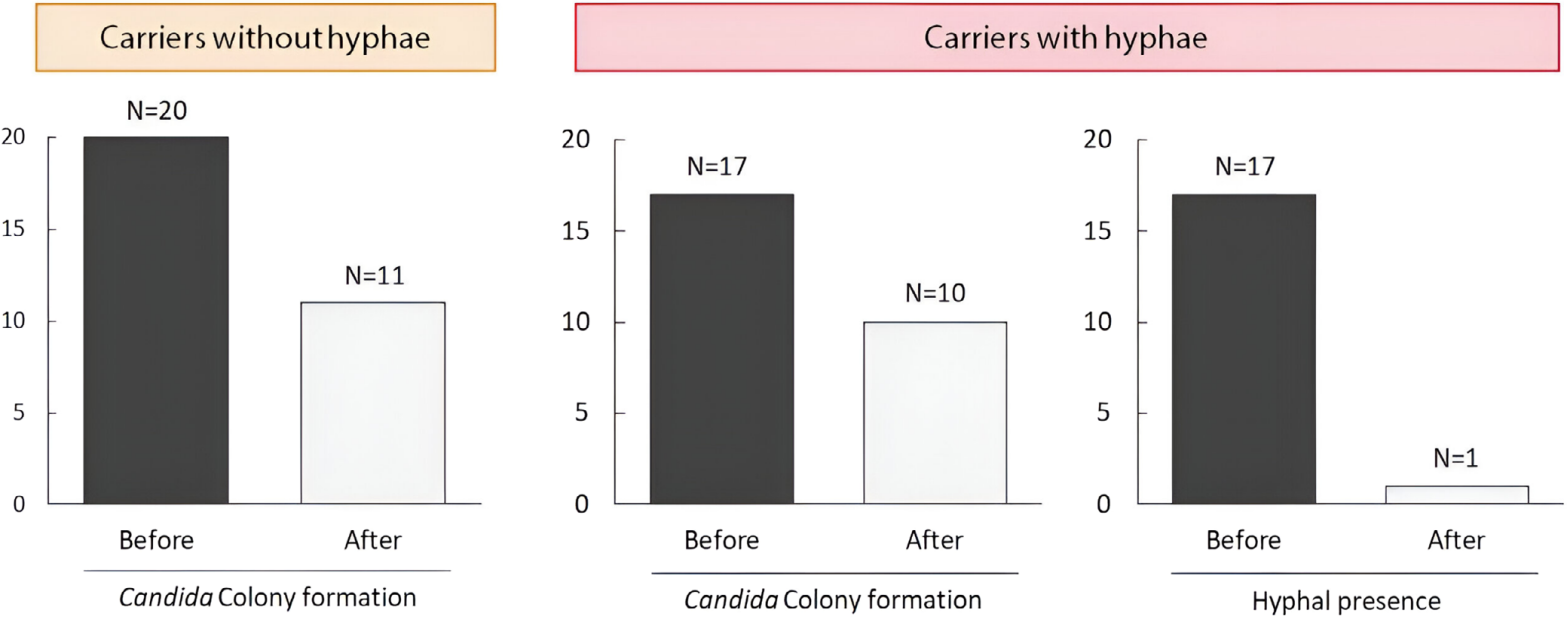
Changes in *Candida* colony formation and hyphal presence before and after antifungal treatment. Bar graphs depicting the proportions of participants with detectable *Candida* colonies and hyphal forms in tongue biofilms before and after 2 weeks of topical fluconazole treatment resulted in a substantial reduction in hyphal presence, particularly in carriers with hyphae, whereas decreases in overall colony formation were more modest.

Following topical fluconazole administration, changes in colony formation and hyphal presence were assessed. Treatment with antifungal agent resulted in a marked reduction in hyphae (93.8%) among carriers with hyphae, whereas the decrease in colony formation was modest (45.0% in carriers without hyphae and 37.5% in carriers with hyphae; Figure 3).

### 3.4 Changes in Taste Sensitivity Before and After Antifungal Treatment

Following topical fluconazole administration, changes in taste sensitivity were observed in both carrier groups. Carriers without hyphae showed a significant improvement only in their sensitivity to bitter tastes (*p* = 0.012). In contrast, carriers with hyphae displayed significant improvements in sweet (*p* = 0.002), salty (*p* = 0.007), bitter (*p* = 0.008), and umami (*p* = 0.008) tastes, except sour. Notably, changes in sensitivity for salty (*p* = 0.015) and umami (*p* = 0.034) tastes were significantly greater in carriers with hyphae than in those without hyphae (Table 4).

**TABLE 4:** Mean differences in taste sensitivity before and after topical fluconazole treatment in the carrier group.

	Carriers without hyphae (*N* = 20)		Carriers with hyphae (*N* = 17)		*p* ^‡^
	Mean ± SD	*p* ^†^	Mean ± SD	*p* ^†^	
Sweet	0.45 ± 1.15	0.095	0.47 ± 0.51	0.002	0.946
Sour	0.15 ± 0.59	0.267	0.29 ± 0.69	0.096	0.495
Salty	−0.10 ± 0.72	0.541	0.47 ± 0.62	0.007	0.015
Bitter	0.55 ± 0.89	0.012	0.53 ± 0.72	0.008	0.939
Umami	0.05 ± 0.69	0.748	0.59 ± 0.80	0.008	0.034
Total score	1.15 ± 2.50	0.053	2.35 ± 1.66	< 0.001	0.099

Abbreviation: Mean±SD, Mean ± standard deviation.

^†^ Paired *t*‐test.

^‡^ Independent *t*‐test.

## 4 Discussion

Taste dysfunction is a hallmark symptom of OC; however, its precise pathogenesis remains elusive. We hypothesized that either the presence of *C. albicans* or, more specifically, its hyphal phenotype may contribute to impaired taste sensitivity, a notion that is significant given the opportunistic nature of *Candida* infection and its diverse clinical phenotypes [9, 16]. Biofilm formation, which involves the transition from yeast to hyphal forms, is a key factor contributing to *Candida* pathogenicity and variability in OC presentation [24, 25, 26].

Previous studies have investigated the relationship between *C. albicans* and taste disorders by comparing taste sensitivity among non‐carriers, healthy carriers, and patients with tongue candidiasis. These studies demonstrate that *C. albicans* carriage, even without clinical candidiasis, is associated with altered taste perception. Additionally, antifungal treatment has been shown to improve gustatory function in both symptomatic and asymptomatic carriers, suggesting a direct link between *C. albicans* and taste dysfunction [18]. However, the underlying mechanisms, particularly the roles of biofilm formation and hyphal transition, remain unclear.

Our study analysed both *Candida* carriage and smear tests to assess tongue biofilms, allowing a more precise evaluation of *C. albicans* colonisation. By stratifying participants into non‐carriers, carriers without hyphae, and carriers with hyphae, we aimed to determine the distinct role of hyphal transition in taste dysfunction. Our findings provide new insights into the clinical impact of biofilm‐associated hyphal forms, as biofilms are known to compromise epithelial integrity and interfere with taste stimulus transmission [5, 8, 27]. Notably, hyphal forms were consistently present whenever a colony was detected in culture, reinforcing their association with active disease progression and mucosal invasion.

Our results demonstrated that both unstimulated and stimulated salivary flow rates were significantly lower in *Candida* carriers than in non‐carriers, with particularly robust differences observed for stimulated saliva, as confirmed by post hoc analyses. This finding aligns with previous evidence that hyposalivation is a well‐known risk factor for OC, with *Candida* carriage frequently observed in individuals with xerostomia [28, 29, 30]. Because saliva contains antifungal proteins, antibodies, and other innate immune components that inhibit *Candida* adhesion and colonisation [28, 29], reduced salivary flow may predispose individuals to persistent biofilm formation. However, the distinct roles of unstimulated and stimulated saliva in biofilm formation remain unclear. Variations in the molecular composition between unstimulated and stimulated saliva highlight the potential for differing roles in *Candida* colonisation and biofilm dynamics [31]. This observation underscores the need for further research.

Taste sensitivity assessments revealed that carriers exhibited reduced sensitivity to sweet and bitter tastes, with an additional significant decline in sensitivity to umami taste in carriers with hyphae. These findings suggest that *C. albicans* colonisation may impair taste function even in the absence of overt infection, while the presence of hyphal forms may contribute to more severe dysfunction via multiple mechanisms. A possible explanation for this phenomenon is interference with taste signal transmission caused by the development of biofilms by *Candida*. These structured communities of yeast and hyphal cells form a physical and biochemical barrier that impedes effective contact between taste stimuli and receptors on the lingual epithelium [17]. This obstruction may be particularly relevant for umami perception, which relies on the detection of amino acids and nucleotides and requires effective interactions with the receptor sites.

Beyond physical obstruction, biofilm‐associated hyphal forms mediate taste dysfunction through enhanced epithelial disruption, immune activation, and interference with molecular signalling. Hyphae are known to penetrate mucosal layers, forming intercellular gaps and facilitating deeper invasion into host tissues [5, 32]. This process triggers inflammatory responses characterised by the upregulation of proinflammatory cytokines such as interleukin (IL)‐1β, IL‐6, and IL‐17, which influence taste bud cell turnover and may disrupt peripheral neural signalling [33, 34, 35]. Virulence factors, including candidalysin and Als3, play a central role by activating mitogen‐activated protein kinase (MAPK) pathways particularly p38 and EGFR‐ERK through interaction with epithelial receptors such as epidermal growth factor receptor (EGFR) and ephrin type‐A receptor 2 (EphA2), amplifying mucosal injury and inflammatory signalling [36, 37]. Pattern‐recognition receptors, such as TLR2, TLR4, and Dectin‐1/2, expressed on gustatory and epithelial cells, recognise Candida antigens and contribute to innate immune activation [34]. These pathways may modulate taste perception by altering ion channel activity (e.g., ENaC), promoting apoptosis of taste bud cells, and impairing neural signal transduction [35]. Additionally, inflammation‐induced changes in the tongue's microenvironment including shifts in local pH, salivary protein composition, and immune effector activity may further compromise epithelial integrity and receptor sensitivity, compounding the effects of biofilm formation and hyphal invasion on gustatory function [38, 39]. While our study design could not isolate the individual contributions of these factors, future mechanistic studies should delineate the specific roles of pH alteration, virulence factor signalling, and physical obstruction in hyphae‐associated dysgeusia.

Following topical fluconazole treatment, taste sensitivity significantly improved, particularly in carriers with hyphae. This clinical recovery underscores the pathogenic relevance of hyphal forms and supports their role as therapeutic targets in OC‐associated dysgeusia. The marked improvement following antifungal therapy targeting biofilm‐associated hyphal forms reinforces their role in driving taste dysfunction and aligns with prior evidence that eradication of hyphal structures restores taste perception [18]. This further supports the hypothesis that the presence of hyphae, rather than *C. albicans* carriage alone, is a significant factor contributing to taste impairment. Notably, although fluconazole effectively reduced the hyphal forms, its impact on overall Candida colony formation was less pronounced, indicating that biofilm‐associated hyphae may be the primary driver of taste dysfunction, rather than mere fungal presence. Consequently, antifungal treatment strategies targeting biofilm‐associated hyphal forms are essential, and early intervention in hyphae‐positive individuals may prevent progression to persistent taste disturbances [18, 40]. These results underscore the importance of differentiating between colonisation and active hyphal invasion when evaluating candidiasis associated taste disturbance.

Our study had several limitations. First, although our initial aim was to investigate Candida hyphae in healthy subjects, the opportunistic nature of Candida meant our cohort necessarily included individuals with heterogeneous comorbidities and medication histories, which may have affected our findings. Second, despite efforts to minimise confounding from oral lesions, the influence of other oral pathologies on taste perception could not be entirely excluded. Third, the absence of post‐treatment measurements of salivary flow limited our ability to determine whether the observed improvement in taste sensitivity following antifungal therapy was independent of salivary changes. Finally, our taste assessment methods did not incorporate more advanced techniques, such as trimatches, which might have provided further nuances in evaluating taste sensitivity.

Nevertheless, our study is the first to explore the relationship between the presence of *C. albicans* , particularly its hyphal phenotype, and taste dysfunction in conjunction with changes in salivary flow, thereby offering valuable insights into the pathogenesis of taste dysfunction in OC. In particular, we demonstrated that the hyphal transition of *C. albicans* is associated with clinically observable taste impairment, highlighting its potential clinical relevance as a diagnostic and therapeutic marker beyond mere fungal presence. Future research should explore how hyphal invasion of the oral epithelium contributes to taste dysfunction using advanced histopathological and molecular approaches and whether antifungal treatment can restore salivary flow and enhance taste perception. In particular, studies utilising transcriptomic or immunohistochemical techniques could help elucidate how Candida‐induced inflammation influences the expression and function of taste receptors and downstream signalling molecules. Additionally, in vitro or in vivo models can be used to evaluate specific interactions between fungal virulence factors and host epithelial pathways.

Our findings suggest that *C. albicans* carriage, especially when accompanied by hyphal forms, is associated with reduced salivary function and impaired taste sensitivity. The marked improvement in taste perception following antifungal treatment, particularly in the hyphae‐positive group, suggests that hyphal invasion plays a critical role in the pathogenesis of taste dysfunction in patients with OC. Collectively, these results underscore the importance of early identification and management of hyphal invasion to prevent persistent or long‐term taste disturbances associated with OC.

## Author Contributions

**Younjung Park:** conceptualization, data curation, methodology, resources, writing – original draft, validation. **Hyo‐Jung Jung:** conceptualization, data curation, investigation, methodology, writing – original draft, formal analysis. **Sin Hye Hwang:** investigation, writing – original draft. **Eunae Sandra Cho:** writing – review and editing, resources. **Hyung‐Joon Ahn:** conceptualization, project administration, supervision, writing – review and editing.

## Funding

This work was supported by the National Research Foundation of Korea.

## Ethics Statement

This study was conducted at Yonsei University Dental Hospital between February 2021 and September 2022 in accordance with the principles of the Declaration of Helsinki. The study protocol was reviewed and approved by the Institutional Review Board of Yonsei University Dental Hospital (IRB No. 2–2020‐0046). The authors confirm that the ethical policies of Mycoses, as outlined in the journal's author guidelines, have been adhered to.

## Conflicts of Interest

The authors declare no conflicts of interest.

## Data Availability

The data that support the findings of this study are available on request from the corresponding author. The data are not publicly available due to privacy or ethical restrictions.
